# A Bayesian model to estimate the cutoff value of TSH for management of preterm birth

**DOI:** 10.1371/journal.pone.0283503

**Published:** 2023-03-29

**Authors:** Maryam Rahmati, Sima Nazarpour, Sonia Minooee, Samira Behboudi-Gandevani, Fereidoun Azizi, Fahimeh Ramezani Tehrani

**Affiliations:** 1 Reproductive Endocrinology Research Center, Research Institute for Endocrine Sciences, Shahid Beheshti University of Medical Sciences, Tehran, Iran; 2 Department of Midwifery, Varamin-Pishva Branch, Islamic Azad University, Tehran, Iran; 3 Centre for Midwifery, Child and Family Health, Faculty of Health, University of Technology Sydney, Sydney, NSW, Australia; 4 Faculty of Nursing and Health Sciences, Nord University, Bodø, Norway; 5 Endocrine Research Center, Research Institute for Endocrine Sciences, Shahid Beheshti University of Medical Sciences, Tehran, Iran; University of Campinas, BRAZIL

## Abstract

**Background:**

Determining a thyroid hormone cutoff value in pregnancy is challenging issue and several approaches have been introduced to optimize a utility function. We aimed to estimate the cutoff value of TSH using Bayesian method for prediction of preterm-birth.

**Methods:**

This study was a secondary-analysis of the population-based data collected prospectively within the framework of the Tehran Thyroid and Pregnancy Study. A total of 1,538 pregnant women attending prenatal clinics.

**Results:**

Using Bayesian method resulted a TSH-cutoff of (3.97mIU/L,95%CI:3.95–4.00) for distinguishing pregnant women at risk of preterm-birth. The cutoff was associated with acceptable positive predictive and negative predictive values (0.84,95% CI:0.80–0.88) and 0.92 (95%CI: 0.91–0.94), respectively). In women who were negative for thyroid peroxides antibody (TPOAb) with sufficient urinary iodine concentration (UIC), the TSH cutoff of 3.92 mIU/L(95%CI:3.70–4) had the highest predictive value; whereas in TPOAb positive women with insufficient UIC, the cutoff of 4.0 mIU/L(95%:CI 3.94–4) could better predict preterm birth. Cutoffs estimated in this study are close to the revised TSH value of 4.0mIU/L which is currently recommended by the American Thyroid Association.

**Conclusion:**

Regardless of TPOAb status or iodine insufficiency, risk of preterm labor is increased in pregnant women with TSH value of > 3.92 mIU/L; these women may benefit from Levothyroxine (LT4) therapy for preventing preterm birth.

## Introduction

Preterm birth is a leading cause of neonatal morbidity and mortality worldwide [1]. In recent years, researchers have attempted to develop reliable prediction models using the potential risk factors to predict and prevent preterm birth [2]. Of different risk factors, the role of overt thyroid dysfunction in increasing the risk of preterm birth is well established. However, data is inconsistent regarding milder thyroid dysfunctions [3].

Subclinical hypothyroidism (SCH), characterized by elevated thyrotropin (TSH) and normal free thyroxine, is the most common thyroid dysfunction during pregnancy [4]. The vast majority of studies have reported the association between SCH and perinatal complications such as pregnancy loss, hypertensive disorders and preterm birth [5–10]. There are other reports, however, which have not supported this association [11]. Compared to other outcomes, the relationship between preterm birth and SCH appears to be less controversial, since many recent observational studies and clinical trials have proven the benefit of Levothyroxine (LT4) therapy in preventing preterm birth [3, 12–14].

The first challenge in SCH in pregnancy is the definition of normal TSH range [15]. Based on the 2017 American Thyroid Association (ATA) guideline, the initial TSH cutoff value of 2.5 mIU/L for the first trimester of pregnancy has been revised to a higher upper reference range of 4.0 mIU/L [3]. Using the new cutoff, the association between SCH and preterm birth appears to become stronger compared to using the previous cutoff of 2.5 mIU/L [12]. However, the exact predictive cutoff is still a matter of debate across studies [15].

The second challenge regarding the determination of TSH cutoff, is the interrelation between thyroid function tests and other thyroid-related parameters such as thyroid peroxides antibody (TPOAb) and maternal iodine status. Evidence indicates that the association between SCH and preterm birth may be dependent on the TPOAb positivity, suggesting that in the absence of TPOAb, the TSH> 4.0 mIU/L may not result in preterm birth [13]. This is an important evidence in terms of initiation of treatment in pregnancy. Based on the ATA’s recommendation, TSH cutoffs should be determined in both euthyroid and TPOAb negative women [3]. There are also reports which have identified maternal iodine status as a predictor of adverse pregnancy outcomes in SCH [16, 17]. However, currently, there is no cutoff available to predict these outcomes by considering the thyroid autoimmunity and iodine status of women. Therefore, estimating a TSH cutoff based on these parameters will enable clinicians to provide a more individualized clinical approach for women.

Another issue in determining TSH cutoff is regarding the statistical methods. Determining a cutoff value in continuous diagnostic tests is challenging and several approaches have been introduced to optimize a utility function [18, 19]. The receiver operating characteristics (ROC), is the most common method which plots sensitivity versus 1-specificity [20, 21]; however, it does not provide information on the diagnostic accuracy of a test at an individual level [22], which is crucial for clinical decision making. Using an appropriate statistical modeling such as the Bayesian method will enable us to overcome the limitations of previous approaches [19] and can precisely categorize each individual pregnant woman who is at risk of adverse outcome.

In this study, we used the population-based data collected in the Tehran Thyroid and Pregnancy Study (TTPS), to estimate TSH cutoffs for prediction of preterm birth, considering TPOAb status and urinary iodine concentration (UIC) of subgroups of women.

## Material and methods

### Study design and participants

This study is a secondary analysis of data collected in the Tehran Thyroid and Pregnancy Study (TTPS), a two-phase single blinded population-based randomized clinical trial on pregnant women who lived in Tehran. The details of the study have been reported previously [23]. Briefly, in the first phase of TTPS, a total of 2,406 pregnant women from among those receiving prenatal care in centers under coverage of Shahid Beheshti University of Medical Sciences, were screened for thyroid dysfunction in the first trimester. For this purpose, overnight fasting blood samples were collected at the first prenatal visit to measure serum levels of TSH, thyroxin (T4), T- uptake (RTU), and thyroid peroxidase antibody (TPOAb), and the thyroid status of pregnant women were identified, and TPOAb <50 IU/mL were considered euthyroid TPOAb negative. Participants were also asked to collect three casual morning urine samples (5–10 mL) on an every other day basis for measurement of urinary iodine concentration (UIC) to calculate mean UIC (MUIC). Spot urine samples were collected and stored at −20°C. We defined iodine deficiency as MUIC < 150 μg/L as suggested by the joint task force of the World Health Organization (WHO), the United Nations Children’s Fund (UNICEF), and the International Council for the Control of Iodine Deficiency Disorders (ICCIDD) [24]. Subclinical hypothyroidism was defined as normal FT4I (1–4.5), despite elevated TSH (2.5–10 μIU/mL).

Women with SCH, regardless of their TPOAb status, were invited to participate in the second phase of the study and were randomly assigned into either of the following groups 1) treatment with LT4 or 2) without treatment. Euthyroid TPOAb ngative women served as the healthy controls and were followed up until delivery. For the purpose of this study, we excluded pregnant women with twin pregnancies (n = 29), those with overt hypo/hyperthyroid (n = 102), those who had used LT4 during pregnancy (previous consumers or those selected as interventional group in second phase of the study (n = 286), and those with missing data (n = 277). Finally a total of 1,538 pregnant women were included. The primary outcome of this study was preterm birth that is defined as babies born alive before 37 weeks of gestation [25].

### Laboratory assessments

T4 was measured by radioimmunoassay (RIA) using commercial kits (Izotop Kit, Budapest co, Hungary) and TSH was measured by immunoradiometric assay (IRMA) using the Gamma-counter (Dream Gamma- 10, Goyang-si, Gyeonggi-do, South Korea). TPOAb and RTU were measured by immunoenzymometric assay (IEMA) (Monobind Kit, Costa Mesa, CA, USA) and enzyme immunoassay (EIA) (Diaplus Kit, San Francisco, CA, USA), respectively, using a calibrated ELISA reader (Sunrise, Tecan Co. Salzburg, Austria). Intra- and inter-assay coefficients of variation (CV) for T4, TSH, RTU, and TPOAb were 1.1% and 3.9%, 1.9% and 4.7%, 2.2% and 4.3% and 1.0% and 1.6%, respectively. Since free T4 immunoassays may be affected by changes of serum thyroxine-binding globulin and albumin during pregnancy, Free Thyroxine Index (FT4I) was used. UIC was determined using a manual method, based on the Sandell–Kolthoff technique. The intra-assay CVs in three ranges of 3.4, 12.5 and 37.1 μg/L were 8.5, 7.2 and 9.6%, respectively, and inter-assay CVs % were 9.1, 8.6 and 12.3%, respectively.

The study was approved by the ethics committee of the Research Institute of Endocrine Sciences (RIES); approval no: IR.SBMU.ENDOCRINE.REC.1397.273.

### Statistical analysis

Continuous variables were checked for normality using the one-sample Kolmogorov-Smirnoff test; categorical variables were expressed as number and percentages. Continuous variables with normal distribution were expressed as mean (standard deviation) and those with Non-normal distribution were expressed as median (interquartile).

In order to estimate the posterior distribution of cutoff value for TSH as a biomarker for preterm birth as well as its predictive values, we applied a Bayesian method. This method introduced by Vradi et al., assumes that X = (*X*_1_, *X*_2_, …, *X*_*n*_) ∈ ℝ is the continuous biomarker measurement for n individuals that can be measured on all patients. It also defines *Y* = (*Y*_1_, *Y*_2_, , *Y*_*n*_) as a binary response variable, where *Y*_*i*_∈{0,1} for all i = 1,…,n; such a way that Y_i_ = 0 denotes the women without preterm birth and Y_i_ = 1 the responder subjects or women with preterm birth. The probability of response p is modeled by a step function in terms of positive predictive value (PPV) and negative predictive value (NPV) of the biomarker assay [18, 19].


$$Y|X\sim Bernoulli\left(p\right)$$

$$p\left(x\right)=P\left(Y=1|X=x\right)=\left\{\begin{array}{c} p_{1}=P\left(Y=1|X\leq cp\right)\\ p_{2}=P\left(Y=1|X>cp\right) \end{array}\right.\begin{array}{c} ,\\ , \end{array}\;\begin{array}{c} for\;x\leq \;cp\\ for\;x>\;cp \end{array}.$$

In this study Y is defined as preterm birth status, X as TSH measurement and cp as a cutoff value for TSH. Therefore p_1_ = 1-NPV expresses the probability of preterm birth given TSH is below the cutoff value cp and p_2_ = PPV expresses the probability of preterm birth given that TSH is greater than cp. The parameters p1, p2 and the cutoff are assumed to have probability distributions reflecting the uncertainty in their parameters values. Among possible distributions for probabilities of response p1 and p2 we considered the simplest case i.e. uniform priors so that PPV>1-NPV; *p*_1_~*Unif* (0, *p*_2_) and *p*_2_~*Unif* (0,1), i.e. by choosing p2~Unif(0,1) we were interested in the posterior distribution of the cutoff expected to yield a PPV between 0% and 100%.

Moreover, for the cutoff cp a weighted sum of informative and non-informative priors have been considered to acknowledge potential prior-data conflict and allow for robustness. The probability density function (pdf) of the uninformative component and the pdf for the informative part were defined cp1 and cp2, respectively. We decided to choose uniform distribution that the support set was the interval (2,4) for informative part because of the initial TSH cutoff value of 2.5 mIU/L and the new cutoff 4.0 mIU/L. Non-informative prior (cp1) for TSH cutoff was considered as a uniform distribution in interval (0,10) [19].


$$cp=w\;\;cp_{1}+\left(1-w\right)cp_{2}$$

$$cp_{1}\sim Unif\left(0,10\right)$$

$$cp_{2}\sim Unif\left(2,4\right)$$

$$w\sim Unif\left(0,1\right)$$

We compared the cutoff obtained from the Bayesian approach with the predictive summary index (PSI) which is a frequentist approach. It estimates the optimal cutoff by maximizing the difference in predictive values for all possible cutoffs and is expressed as *PSI* = *max*_*cutoff*_{*PPV*_*cutoff*_ + *NPV*_*cutoff*_− 1}. For the latter approach, the confidence intervals were calculated by the bootstrap method. For the Youden index and PSI, the package “OptimalCutpoints” was used in R version 3.6.1.

We repeated Bayesian analysis for estimating the cutoff values for TSH measurement in two subpopulations: 1) subjects with negative TPOAb and sufficient UIC, 2) subjects with positive TPOAb and insufficient UIC.

Estimation of the model parameters was carried out using the Markov chain MonteCarlo algorithm [26], which is a freely available program called WinBUGS 1.4. The burn-in consisted of 10,000 iterations; 50,000 subsequent iterations were used for posterior summaries. Convergence with a two-chain run was judged by using plots and Gelman and Rubin’s [27] measure of scale reduction factor which was found to lie between 0.9 and 1.2.

## Results

Characteristics of women who participated in the study, are presented in Table 1. The average age (years), BMI (kg/m^2^) and gestational age (wk) of participants at the initiation of the study were 27.3 (5.2), 25.1 (4.4) and 11.7 (5.1), respectively. Of a total 1,538 women, 79 (5.1%) were positive for TPOAb, and 1459 (94.9%) were negative TPOAb. 491 (31.9%) women had sufficient UIC and 594 (38.6) were found to have insufficient UIC. Data for urine iodine were missing in 453 (29.5%) of participants, and no imputation method was done for missing data. Preterm birth occurred in 126 women (8.2%).

**Table 1:** Characteristics of the study participants.

Characteristics		Total pregnant women (n= 1,538)	Euthyroid women (n=1018)	Pregnant women with negative TPOAb and sufficient UIC (n=479)	Pregnant women with positive TPOAb and insufficient UIC (n=41)
Age (yr) ^a^		27.3±5.2	27.5±5.2	27.1±5.2	25.9±3.9
Body mass index (kg/m^2^) ^a^		25.1±4.4	25.3±4.5	24.9±4.2	23.9±3.6
Parity ^c^	Primipara	571 (37.1)	377 (37.0)	178 (37.2)	16 (39.0)
	Multipara	967 (62.9)	641 (63.0)	301 (62.8)	25 (61)
Gestational age at first visit (wk) ^a^		11.7±5.1	11.8±5.2	11.6±5.1	11.6±4.8
TSH (mIU/L) ^b^		1.6 (0.9-2.3)	1.6(0.9-2.2)	1.6(0.8-2.1)	4.3(2.4-6.9)
UIC (μg/l) ^b^ ^¥^		137.7 (90.1-223.1)	94.3(68.5-120.2)	234.2(185.5-279.9)	103.2(75.9-123.0)
TPOAb (IU/ml) ^b^		4 (2-9)	5(3-10)	4(2-8)	127(70-210)
Preterm birth ^c^		126 (8.2)	90 (8.8)	32(6.7)	4(9.8)

TSH: thyroid stimulating hormone; UIC: urinary iodine concentration; TPOAb: thyroid peroxidase antibody

a Mean ±standard deviation; b Median (inter-quartile range); c n (%)

¥ Available data for these variables were 1,085.

**Fig 1:**
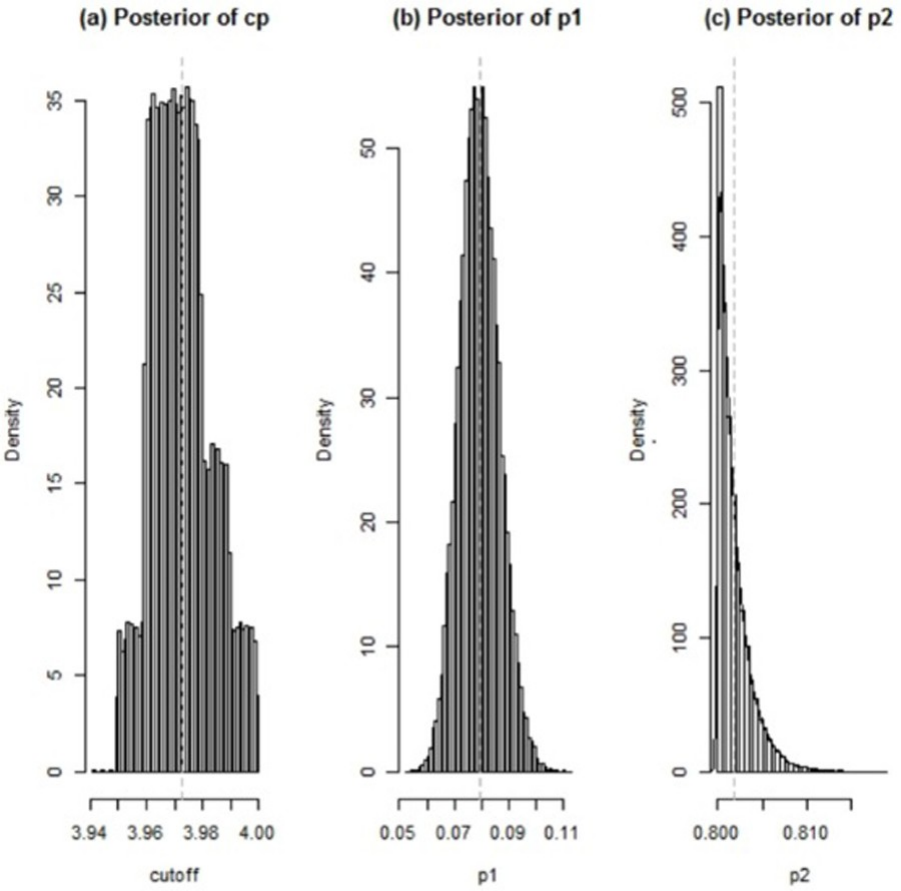
Plot of the posterior distribution for the parameter cp (a), p_1_ (b), p_2_ (c) estimated by the Bayesian model. The vertical lines denote the median of the distribution.

The posterior distributions for the cutoff (a) and the predictive values p_1_ and p_2_ (b,c) are shown in Fig 1. The posterior mean of the cutoff was 3.97 with 95% credible interval (95% CI) of 3.95, 4.0. At that cutoff, the Bayesian posterior means for PPV and 1-NPV (95% CI) were found to be 0.84 (0.80, 0.88) and 0.08 (0.06, 0.09), respectively (Table 2).

**Table 2:** Cutoff, PPV and 1-NPV estimated by Bayesian and frequentist approaches with 95% credible/confidence intervals.

	Total pregnant women (n = 1,538)			Pregnant women with negative TPOAb and sufficient UIC (n = 479)	Pregnant women with positive TPOAb and insufficient UIC (n = 41)
**Parameter**	Bayesian method Estimate (95% CI)	Youden Estimate (95% CI)	PSI Estimate (95% CI)	Bayesian method Estimate (95% CI)	Bayesian method Estimate (95% CI)
**Cutoff**	3.97 (3.95, 4.0)	3.14 (3.01, 3.22)	9.17 (8.42, 9.38)	3.92 (3.70,4.0)	4.0 (3.94,4.0)
**PPV**	0.84 (0.80, 0.88)	-	0.5 (0.21, 0.79)	0.78 (0.71,0.82)	0.69 (0.65,0.73)
**1-NPV**	0.08 (0.06, 0.09)	-	0.08 (0.02, 0.42)	0.12 (0.08,0.15)	0.14 (0.09,0.21)

PPV: positive predictive value; 1-NPV: 1-negative predictive value; CI: confidence interval; PSI: predictive summary index

**Fig 2:**
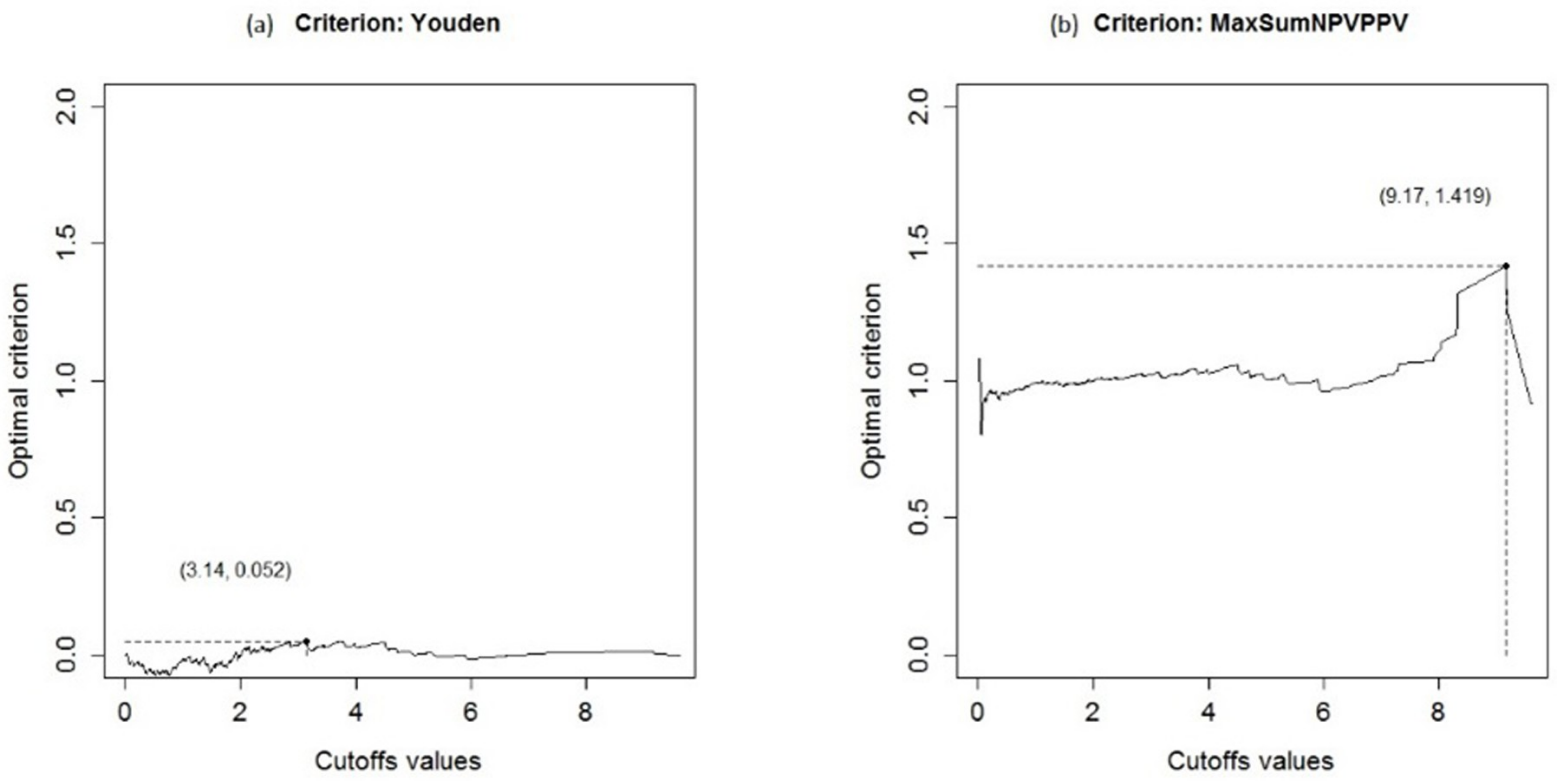
The receiver operating characteristic (ROC) curve of the participants in this study.

The optimal cutoff was estimated as 3.14 by the Youden index and the area under the ROC curve (AUC) was estimated 0.5 (95% CI 0.43, 0.54) that results in uninformative test. Based on the PSI method, the cutoff was estimated as 9.17 (Fig 2 and Table 2). For the PSI, PPV and 1-NPV were equal to 0.50 (95% CI 0.21, 0.79) and 0.08 (95% CI 0.02, 0.42) respectively.

**Fig 3:**
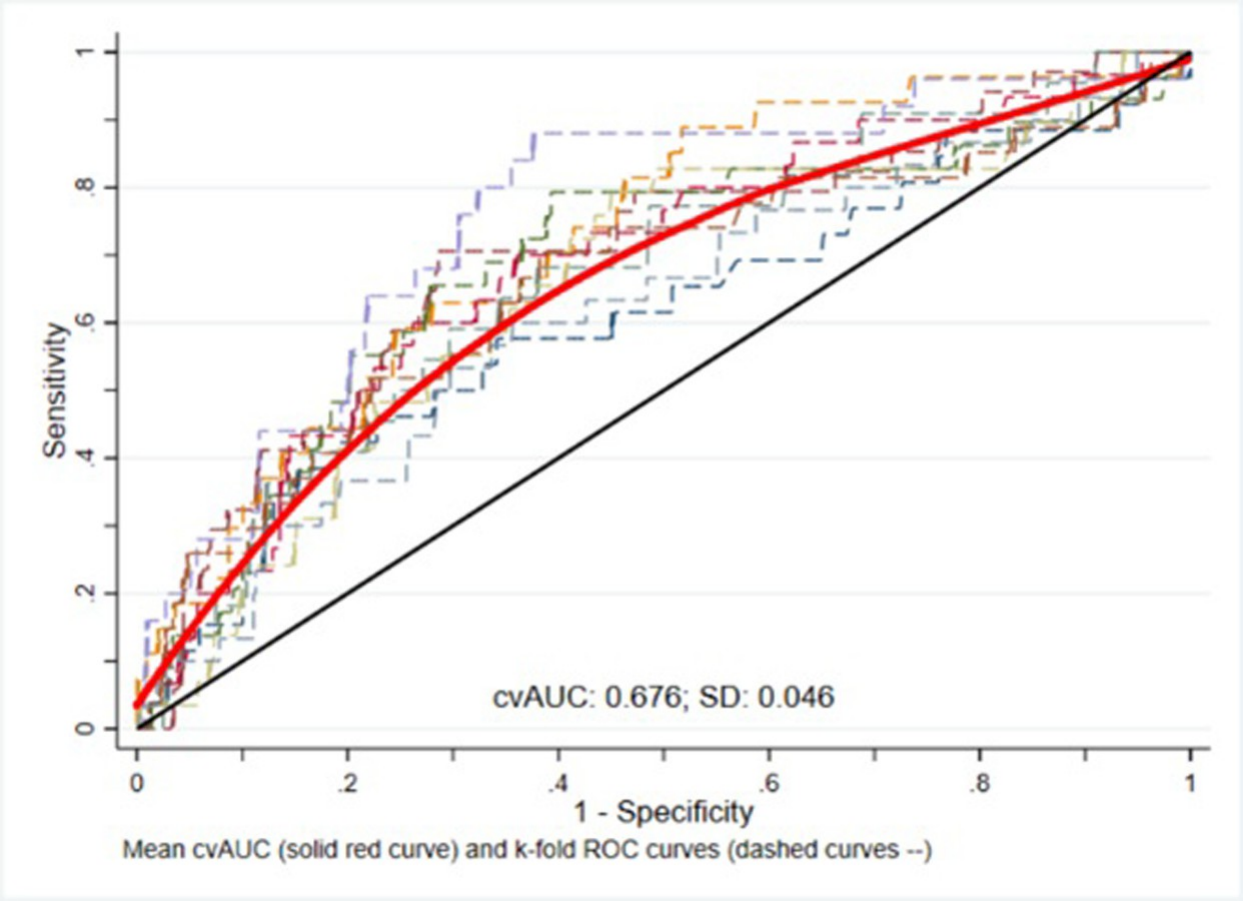
10-fold cross validation plot to validate cutoff in total population.

To confirm the validity of the obtained results, we used split-sample validation method and randomly split data into two samples: 70% = training sample, and 30% = validation sample. We applied data contained in the training set to the Bayesian model and obtained the cutoff value of 3.86 with 95% CI: (2.28,3.97), then run a Bayesian logistic regression model with this cutoff on validation set, averaging the AUCs (area under curve) corresponding to each fold, and bootstrapping the cross-validated AUC to obtain statistical inference and 95% confidence intervals. We plotted K-fold AUC and cross-validated AUC. The AUC calculated using cross-validation method shows 0.01 points lower accuracy (AUC = 0.676) than the AUC computed using the classical approach from the predicted probabilities of preterm delivery (AUC = 0.686), which shows the ability of the Bayesian model to generalize to new cases (Fig 3).

The posterior mean of the cutoff value for subjects with TPOAb negative and sufficient urine iodine (479) was 3.92 (95% CI 3.70, 4.0). This value for participants with TPOAb positive and insufficient urine iodine (41) was 4.0 (95% CI: (3.94, 4).

Table 3 represents frequency of participants with and without preterm birth by TSH level dichotomized by Bayesian estimated optimal cutoff, for all participants, and each subpopulation.

**Table 3:** Frequency of participants with and without preterm birth by TSH level dichotomized by Bayesian estimated optimal cutoff, for all participants, and each subpopulation.

	Total pregnant women (n = 1,538)		Pregnant women with negative TPOAb and sufficient UIC (n = 479)		Pregnant women with positive TPOAb and insufficient UIC (n = 41)	
Preterm birth	Bayesian cutoff		Bayesian cutoff		Bayesian cutoff	
	≤3.97	>3.97	≤3.92	>3.92	≤4.0	>4.0
**No**	1387(98.6%)	25(19.1%)	436(99%)	11(28.2)	35(100)	2(33.3)
**Yes**	20(1.4%)	106(80.9)	4(1%)	28(71.8)	0	4(66.7)
**Total**	1407(100)	131(100)	440(100)	39(100)	35(100)	6(100)

Analysis was repeated excluding those with induced preterm birth (n = 17); the result did not change.

## Discussion

The present study demonstrated that a TSH cutoff value of 3.97 (95% CI 3.95, 4.0) was able to distinguish pregnant women at risk of preterm birth. This threshold has acceptable PPV and NPV of 0.84 (95% CI 0.80, 0.88) and 0.92 (95% CI 0.91, 0.94), respectively. This threshold is much closer to the revised TSH upper reference range of 4.0 mIU/L that has been endorsed by the latest ATA guideline. We found that the TSH cutoff value resulted by ROC curve is not predictive enough for predicting the risk of preterm birth for individual cases.

Through a large number of systematic reviews and clinical trials in the recent years, the association between SCH and preterm birth has become more clear [14, 28]. Over the past decade, several studies have focused on introduction of TSH cutoff values that are associated with adverse pregnancy outcomes [29–32]. However, there are differences between the values reported due to the genetic background, ethnicity, pregnancy trimester, iodine-intake adequacy and thyroid autoimmunity status of women [3]. In 2011 the ATA proposed a TSH> 2.5 mIU/L in the first trimester as the upper normal value. This threshold has been widely re-evaluated as it may lead to over-diagnosis of thyroid dysfunction in the context of universal screening and that many cases would probably be treated without substantial benefits [33, 34]. Decision on using the optimum TSH cutoff points becomes more complicated when the clinicians need to take into account the thyroid autoimmunity status of women, as it is a known risk factor for preterm birth even in euthyroid women [35, 36].

In TTPS, we have already demonstrated that by using the TSH cutoff 2.5 mIU/L, no significant difference in preterm birth rate was observed between those pregnant women with SCH + TPOAb negative who received LT4 and those without treatment [37]. However using the cutoff of 4.0 mIU/L, we found a significantly lower rate of preterm birth in LT4-treated women, compared with those who received no treatment [37]. We also found that there was no significant difference in terms of preterm birth between those TPOAb positive women with TSH level of <4.0 μIU/mL who received LT4 compared to those who were not treated, but the preterm birth of TPOAb positive women with TSH values of ≥4.0 μIU/mL who received LT4 were significantly lower than those who were not treated [38]. This evidence indicated that the risk of preterm birth in SCH is adjusted through the level of TSH elevation as well as the presence of TPOAb positivity.

Adding to our previous findings, in this study we determined TSH cutoffs by considering the TPOAb status and UIC of women. The cutoff value for TPOAb negativity and sufficient urine iodine was 3.92 (95% CI 3.70, 4.0). For the number of 41 women with TPOAb positivity and insufficient UIC, the Bayesian estimated cutoff was 4.0 (95% CI 3.94, 4.0). The proposed Bayesian method here is very tractable in estimating the parameters of interest, resulting in point estimators that are practically unbiased even for small sample size.

Previous studies have also confirmed the synergistic effects of SCH and TPOAb positivity on poor pregnancy outcomes. Liu *et al*. reported that women with TSH values within the range of ≥2.5 to <5.22 mIU/L did not have an increased risk of pregnancy loss, whereas co-occurrence of high TSH and thyroid antibodies increased the risk of adverse events [39]. Results of a recent meta-analysis by Korevaar et al confirmed the role of TSH> 4.0 mIU/L in increasing the risk of preterm birth in TPOAb positive women [28]. Another recent prospective cohort also found that TPOAb positivity in the absence of TSH elevations does not lead to poor pregnancy outcomes, suggesting that euthyroid women with positive TPOAb are not at risk of preterm birth [40].

Similar to thyroid autoimmunity, the relationship between iodine deficiency and pregnancy outcomes is still controversial and findings are mixed [41, 42]. Iodine is an essential micronutrient for proper thyroid function which its deficiency during pregnancy may affect growth and neurodevelopment of the offspring [43]. Requirement for iodine increases during pregnancy which will result in more pregnant women being classified as iodine deficient [44]. Recent studies have shown that the UIC of euthyroid pregnant women is not associated with poor pregnancy outcomes [45], whereas, the risk of adverse outcomes increases among women with UIC< 100 μg/L, with serum TSH≥ 4.0 μIU/mL [46]. Comparable results from a prospective study among Chinese pregnant women showed that with MUIC of 107.4 μg/L, normal thyroid function was maintained in both mothers and their neonates [41]. However, the authors included only the women who had a spot UIC less than 150 μg/L, had TSH lower than 2.5 mIU/L, and were negative for TPOAb and TgAb. There is also evidence that iodine deficiency in early pregnancy is an independent risk factor for TPOAb positivity [47]. Shi et al reported that both mild iodine deficiency and excessive iodine levels increase the risk of TPOAb positivity in pregnancy which present a U-shaped curve [48]. Based on all these studies, it appears that when high TSH is combined with other parameters such as positive TPOAb and abnormal UIC, different pregnancy outcomes may be expected. As such a cutoff which is purely based on TSH, without considering other thyroid-related parameters may not provide an ideal prediction for pregnancy outcomes.

In SCH, while values of thyrotropin (TSH) increases, level of free thyroxine (Ft4) remains in normal range [49]. These elevations in TSH may be minor and variable across different trimesters of pregnancy, but may adversely affect the maternal and feto-neonatal outcomes. However, debate is ongoing on the screening and treatment of SCH in pregnancy. Importance of having a clear-cut criteria for clinical approach becomes more important in populations which have high rate of SCH such as that of our study [50]. The 2017 ATA guideline strongly recommends treating women with TSH above pregnancy reference range who are TPOAb positive [3]. Weaker recommendations are made for TPOAb negative women. Our previous reports in TTPS as well as reports from other studies have indicated that the effectiveness of treatment is dependent on the timing of the intervention [51, 52]. As such, the earlier diagnosis of at risk women would contribute to a more timely treatment of women.

Evidence is limited on the mechanisms by which thyroid dysfunction and autoimmunity may lead to preterm birth. SCH and/or TPOAb positivity may impair placentation, placental function and fetal growth [53]. Thyroid dysfunction can contribute to abnormal trophoblast invasion and reduced placental vascularity which are important risk factors for preterm birth [54]. Women with either SCH or TPOAb positivity have impaired thyroidal response to hCG stimulation [55, 56] which, in turn, can increase the risk of thyroid dysfunction in pregnancy. Thyroid autoimmunity or hormonal dysfunction can also increase preterm birth rate through immune and infectious pathways [57]. Regardless of the underlying pathway, having access to a tool for prediction of preterm birth will assist clinicians to minimize its adverse outcomes.

An advantage of this study lies in its statistical approach. In previous studies, ROC curve has been the most commonly used method for examining TSH cutoffs [58, 59]. ROC plots the sensitivity versus 1-specificity [20, 21]. Sensitivity and specificity are indeed considered as fundamental operating characteristics of a diagnostic test. However, in practice, ROC does not provide information on the diagnostic accuracy of a test at an individual level [22], which is crucial for decision making of clinicians. Correct classification of a specific person requires information on disease prevalence and PPV and NPV of a test [60]; parameters which have not been provided by the majority of methods.

Sensitivity and specificity have been reported to be misleading in clinical practice, since a high sensitivity does not necessarily imply that a condition is likely given a positive test result [61]. Therefore, positive predictive values and negative predictive values help to put the results of a diagnostic text in clinical context [62].

On the other hand, the Bayesian approach is a way of formulating and dealing with problems where decisions have to be made under a state of uncertainty. This situation is very frequent in problems of medical decision-making [63]. Bayesian methods are particularly attractive tools for diagnostic tests, which are based on established information, usually from related previous studies.

Moreover, in the present study, the idea is to represent the uncertainty about the parameters by a prior distribution using Bayesian approach and we considered PPV and NPV as random variables which can vary around an interval, as a result we are not concern about how likely this study cohort represent the relevant population. In non-Bayesian approach, also, the confidence intervals around the cutoff value must be calculated either by using the delta method or, most commonly, by employing bootstrapping, through the coverage probabilities which can be far from the desired level [19]. In this study, we used Bayesian model which, from among various statistical methods, provides information that can be used for proper categorization at an individual level even with small sample sizes [19]. Moreover, we examined the impact of prior distributions through a sensitivity analysis, however, the results did not change.

Lunceford selected a cutoff on a potentially predictive biomarker that can be used as an enrollment criterion for patient selection. By implementing a Bayesian approach in estimating clinical utility measures, he facilitates cutoff decision-making, but without considering the actual cutoff estimation [64].

However, it should be noted that for determination of the cut-off value, we need to know the pretest probability of the disease of interest as well as the costs incurred by misdiagnosis. This means that even for a certain diagnostic test, the cut-off value is not universal and should be determined for each region and for each disease condition [65].

This study has some further strengths. We used population-based data which is the recommended approach for estimating TSH reference range values [4]. We recruited women who were in their first trimester of pregnancy. Evidence shows that depending on the pregnancy trimesters, the risk of adverse outcomes due to SCH may vary [5], therefore, establishing reference values in the early stages of pregnancy provides an opportunity for early clinical workup and treatment of women who are at risk of preterm birth. Also, a comprehensive thyroid assessment was undertaken including a detailed history, physical examination and thyroid function tests (TSH, T4, T-uptake, TPOAb and UIC). Having said the strengths, results of this study should be interpreted with considering the following limitations. First, the study was conducted among the Iranian population. Ethnicity may influence the serum levels of TSH and thyroid autoimmunity [66]. However it has been shown that ethnicity-specific reference ranges has not improved the diagnosis of SCH [67]. Second, we did not measure anti-thyroglobulin antibodies (anti-Tg); its measurement along with TPOAb could have better differentiated thyroid autoimmune disorder. Third, the casual UIC values may not precisely reflect iodine status of an individual due to day-to-day variability of urinary iodine excretion [68]. Fourth, preterm birth is a broad spectrum word that commonly defined as any birth before 37 weeks completed. Several categorization can be used for preterm birth. It can be sub grouped to three groups according to the weeks of gestation as: extremely preterm (less than 28 weeks), very preterm (28 to 32 weeks), moderate to late preterm (32 to 37 weeks). Preterm infants can also be categorized by birthweight as: < 1000 g: Extremely low birthweight (ELBW) 1000 to 1499 g: Very low birthweight (VLBW) 1500 to 2500 g: Low birthweight (LBW). Preterm birth can also be spontaneous or provider initiated (induced). In present study we have not adequate number of samples for considering subgroups of preterm birth according to the weeks of gestation or birth weight, and only subdivided our subjects based on spontaneous or induced preterm birth. Fifth, our study may be limited by small size of positive groups that could lead to overfitting of our model; however our suggested cut off value seems to be reasonable since it is very close to that obtained by 2017 Guidelines. Furthermore, Bayesian method is very tractable in estimating the parameters of interest, resulting in point estimators that are practically unbiased even for small sample size [19, 63].

To conclude, we found that in our population in the first trimester of pregnancy, the optimal TSH cutoff value for detection of preterm birth in women with SCH is 3.97. Pregnant women with TSH value more than this cutoff value may benefit from Levothyroxine (LT4) therapy for preventing preterm birth. Confirmatory randomized trials are needed to investigate the benefit of LT4 therapy in preventing preterm birth in different subgroups of women based on their TPOAb status and UIC.

## Supporting information

S1 Data (SAV)
